# What Are Physicians' Reasons for Not Referring People with Life-Limiting Illnesses to Specialist Palliative Care Services? A Nationwide Survey

**DOI:** 10.1371/journal.pone.0137251

**Published:** 2015-09-10

**Authors:** Kim Beernaert, Luc Deliens, Koen Pardon, Lieve Van den Block, Dirk Devroey, Kenneth Chambaere, Joachim Cohen

**Affiliations:** 1 End-of-Life Care Research Group, Vrije Universiteit Brussel (VUB) & Ghent University, Brussels, Belgium; 2 Department of Family Medicine and Chronic Care, Vrije Universiteit Brussel (VUB), Brussels, Belgium; 3 Department of Medical Oncology, University Hospital Ghent, Ghent, Belgium; Texas Tech University Health Science Centers, UNITED STATES

## Abstract

**Background:**

Many people who might benefit from specialist palliative care services are not using them.

**Aim:**

We examined the use of these services and the reasons for not using them in a population in potential need of palliative care.

**Methods:**

We conducted a population-based survey regarding end-of-life care among physicians certifying a large representative sample (n = 6188) of deaths in Flanders, Belgium.

**Results:**

Palliative care services were not used in 79% of cases of people with organ failure, 64% of dementia and 44% of cancer. The most frequently indicated reasons were that 1) existing care already sufficiently addressed palliative and supportive needs (56%), 2) palliative care was not deemed meaningful (26%) and 3) there was insufficient time to initiate palliative care (24%). The reasons differed according to patient characteristics: in people with dementia the consideration of palliative care as not meaningful was more likely to be a reason for not using it; in older people their care needs already being sufficiently addressed was more likely to be a reason. For those patients who were referred the timing of referral varied from a median of six days before death (organ failure) to 16 days (cancer).

**Conclusions:**

Specialist palliative care is not initiated in almost half of the people for whom it could be beneficial, most frequently because physicians deem regular caregivers to be sufficiently skilled in addressing palliative care needs. This would imply that the safeguarding of palliative care skills in this regular ‘general’ care is an essential health policy priority.

## Introduction

Evidence is growing that the involvement of specialist palliative care services in the care of people with life-limiting illness results in improved quality of life and quality of dying [1,2]. However, those who might benefit from palliative care services are not always using them[3,4]. Cancer patients in particular more often receive palliative care than those in other illness groups[5,6]. Most research on use of palliative care services is limited to specific illnesses, settings and services and has small sample sizes. Population-based data are necessary to address the public health challenges regarding palliative care. According to the main public health functions outlined by the WHO, these challenges include the assessment and monitoring of the use of and need for palliative care as well as the evaluation of inequalities in the use of palliative care services in order to design public policies of improvement[7].

In line with these challenges, it is important to explore and understand why people are not using these services and why some groups have a better chance of being referred than others[8–10]. Previous explorative qualitative studies have suggested that not using palliative care services may be due to barriers or obstacles to the initiation of palliative care at the level of the patient, the family, the illness, the physician and the health care system[11]. Previous studies evaluating reasons for non-referral to palliative care services have been merely explorative and qualitative and hence do not provide information on the relative importance of the reasons given for not referring, or were based on patient records hence making it impossible to evaluate systematically the reasons given for non-referral beyond basic clinical characteristics.

This large scale population-based retrospective study aims to describe the use of palliative care services and the reasons for not using these services in a population of people who have potentially had palliative care needs before they died based on their underlying cause of death[12]. For people dying from cancer, organ failure, dementia and other life-limiting diagnoses we will address the following research questions: 1) what are the referral rates and timing of referral to palliative care services, 2) what patient characteristics are associated with non-referral, 3) what are the most frequent reasons for not referring people and 4) how are these reasons related to patient characteristics?

## Method

### Study design

We conducted a nationwide death certificate survey in the first half of 2013 based on a large and representative sample of deaths (n = 6871) in Flanders, Belgium. The study design has been repeatedly applied and validated in earlier studies to evaluate end-of-life care decision making[13,14]. The Flemish Agency for Care and Health selected a random stratified sample of all death certificates of persons aged one year or older from January 1st to June 30th 2013. Stratification was disproportionately based on the likelihood that an end-of-life care decision (ELD) had been made, as determined by the cause of death (larger sampling fractions were taken for deaths where an ELD was more likely eg those from cancer).

Every physician certifying a death certificate in the sample was sent a four-page questionnaire about the end-of-life care and decision-making in the corresponding case. The sample was drawn on a weekly basis as new death certificates came in. After data collection a one-page questionnaire was mailed to all non-responding physicians, asking for the reasons for not participating.

### Measurements

The questionnaire asked whether death had occurred ‘suddenly and totally unexpectedly’. If this question was answered negatively–and referral to palliative care would not have been precluded–the physician was asked to answer a number of questions regarding the care received by the patient.

#### Use of palliative care services and timing of referral

The physician was asked whether one or more of the four existing types of palliative care services in Belgium had been involved in the care of the deceased person. These are: multidisciplinary palliative home care teams (multidisciplinary team skilled in palliative care who support the caregivers at home), mobile hospital-based palliative care teams (multidisciplinary team that guides palliative care in the different wards of the hospital), inpatient palliative care units (separate wards in the hospital devoted to palliative care) and a reference person (usually a nurse) trained in and responsible for palliative care in a nursing home.

The physician was also asked to indicate the timing of the referral, i.e. the number of days between the first referral to any of the palliative care services and death.

#### Reasons for not using palliative care services

When no palliative care services had been used, physicians were asked about the reasons why no such services were used: 1) palliative care was not meaningful or not meaningful enough, 2) palliative care was not available, 3) existing care already sufficiently addressed the patient’s palliative and supportive needs, 4) there was not enough time to initiate palliative care, 5) in order not to deprive the patient and/or family of hope, 6) the patient did not want it, 7) the family did not want it or 8) another reason (with the request to specify the reason in text). Existing or regular care is defined in this study as care provided for the patient outside any specialist palliative care service. This can include medical and non-medical care. The reasons in category 8 were afterwards checked by the researchers and allocated to one of the previous categories where possible. Concurrence of more than one reason was possible for each patient. The possible reasons were selected based on relevant literature about factors hindering the use of palliative care services[11,15–18]and on preceding qualitative research on reasons for not using palliative care[19].

Coded demographic and clinical patient characteristics were obtained from the death certificates and were anonymously linked to questionnaire data by the trusted third party: age, sex, place of death, living situation and underlying cause of death coded in three digits ICD-10 codes.

### Data analysis

Data were weighted for disproportionate sampling and for differences due to nonresponse between the response sample and all deaths in terms province of death and place of death (for all other characteristics no response bias was found). After this complex weighting procedure there were no significant differences between response sample and all deaths for the combination of age, sex, marital status, province of death, cause of death and place of death.

For this study we selected a population comprising deaths from underlying causes that, as identified through mixed-methods research based on Rosenwax et al[12], can be considered as indicative of a need for a palliative care approach ie both specialist and non-specialist palliative care. The following underlying causes of death were selected: cancer (ICD-10 C00-C97), organ failure ie heart, renal, liver failure or COPD (ICD-10 J40-47, I11-13, I50, K70-72, N10-12, N18-19), dementia (ICD-10 F01, F03, G30) and other illnesses, ie Parkinson’s disease, motor neurone disease, HIV/aids and non-cancerous neoplasm (ICD-10 D00-48, G20, G12 and B20-24). Any case where dementia was reported as intermediate cause of death or comorbidity was also considered a dementia case. We deliberately chose to do this because dementia as a cause of death is known to be underreported more than other causes of death[20]. Also taking dementia comorbidities into the analysis allows us to give a more representative view of patients living with (and dying with) dementia for whom palliative care is recommended[21]. Persons younger than 18 years were not considered in the analysis. This was only a small group (n = 9) and the palliative care system for children is organized differently from that for adults in Belgium.

Pearson chi square tests analyses for use of palliative care and reasons for not using palliative care services were calculated. We also performed multivariable logistic regression to explore characteristics associated with use of palliative care and reasons for not using palliative care services. The non-parametric Kruskal-Wallis test and multivariable ordinal logistic regression were used to test for differences in time of onset of referral. All analyses are conducted with SPPS 22.0 software using the complex samples function in order to account for the complex survey design (ie disproportionate stratification). All results are weighted.

### Anonymity and ethical considerations

A lawyer was involved in the mailing procedure as trusted third party between responding physicians, researchers and the Flemish Agency for Care and Health to guarantee that completed questionnaires could never be linked to a particular patient or physician. The mailing and anonymity procedures were approved by the Ethical Review Board of the University Hospital of the Vrije Universiteit Brussel, by the Belgian National Disciplinary Board of Physicians, and by the Federal Privacy Commission.

## Results

**Fig 1:**
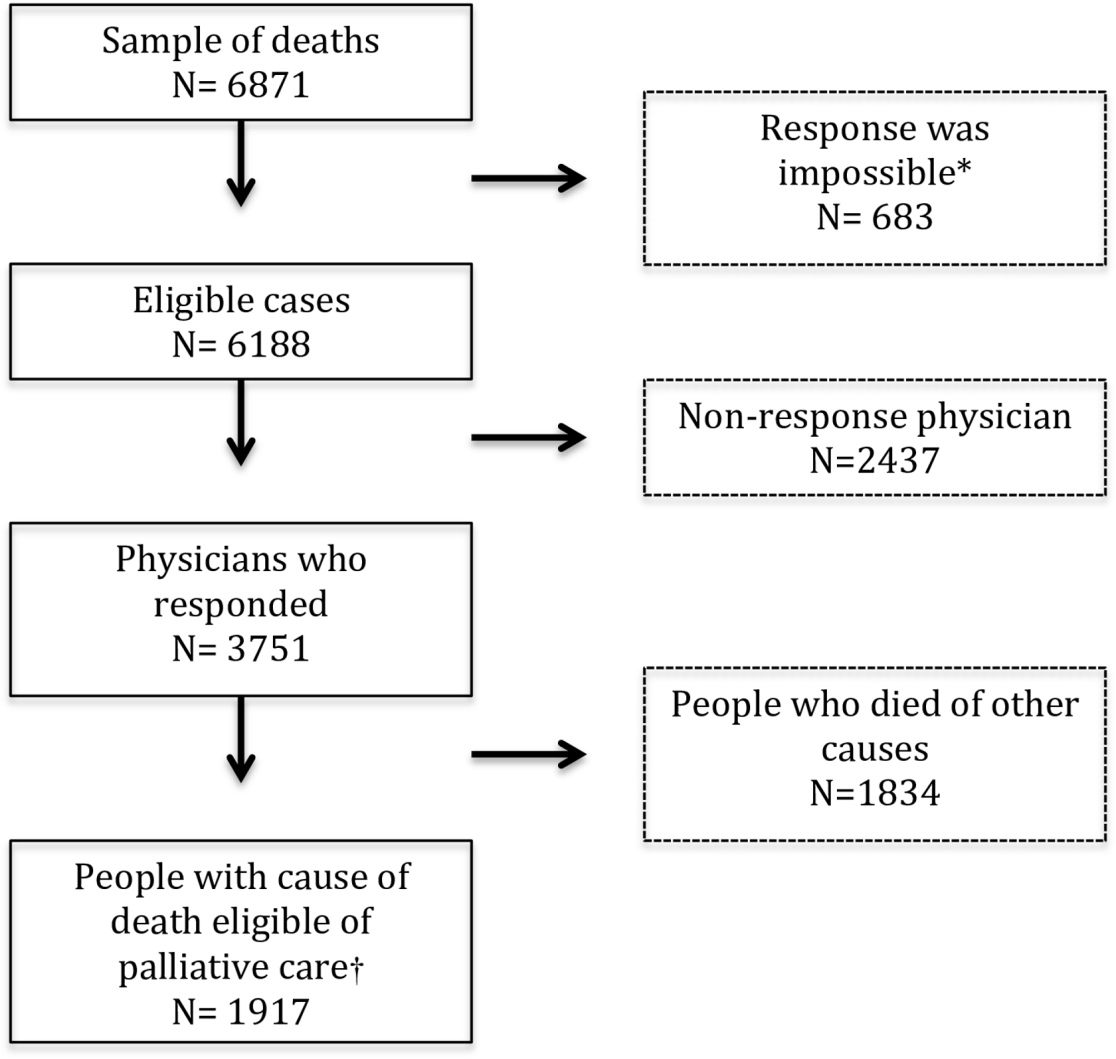
Flow chart of inclusion of deaths and response of physicians. * These cases were deleted from the sample because it was not possible to survey the case. The physician could not identify the decedent with the provided information, no longer had access to the medical file, was not treating physician and did not know who that was, or never received the questionnaire). †For this study we selected a population comprising deaths from underlying causes that, as identified through mixed-methods research based on Rosenwax et al [13] can be considered as indicative of a need for a palliative care approach. The following underlying causes of death were selected: cancer, organ failure, dementia and other illnesses, ie Parkinson’s disease, motor neurone disease, HIV/aids and non-cancerous neoplasm.

Response rate was 60.6%. We obtained a response for 3,751 cases and from the non-response survey we found that response was impossible for 683 deaths e.g. because the physician did not have access to the patient’s medical file or the patient could not be identified (3751/6188 eligible cases) (Fig 1). Of the 3,751 deaths, 1,917 (weighted percentage: 51.1%) were from a cause of death indicative of palliative care need: 25% died from cancer, 12% from organ failure, 12% with dementia and 3% from another illness indicative of palliative care need (Table 1). The causes of death differed significantly in terms of distribution for sex, age, place of death, living situation, and a sudden vs. non-sudden death.

**Table 1:** Characteristics of all deaths and of illness groups considered as indicators for potential benefit of palliative care use (weighted %).

	All deaths	Cancer	Organ Failure	Dementia	Other illness within palliative subset*
Weighted number (weighted % of all deaths)	3751	919 (24.5)	458 (12.2)	436 (11.6)	104 (2.8)
Sex†					
*Male*	50.6	57.1	50.9	36.1	55.2
Age at death†					
*18-64y*	15.4	23.8	10.5	0.9	15.2
*65-84y*	46.6	58.5	42.3	38.6	56.2
*≥ 85y*	38.0	17.7	47.3	60.5	28.6
Place of death†					
*Hospital*	47.5	53.2	45.9	21.4	26.4
*Home*	22.3	33.2	19.0	13.4	31.1
*Nursing home*	26.9	12.4	33.4	63.7	38.7
*Other*	3.2	2.3	1.7	1.8	3.8
Living situation†					
*Alone*	20.6	21.5	21.0	7.9	18.1
*In household with others*	50.2	65.8	43.0	28.3	56.2
*Institution*	28.6	12.1	35.8	63.3	24.8
*Other*	0.6	0.4	0.2	0.5	1.0
Sudden non-sudden deaths†					
*Non-sudden*	61.4	77.9	62.9	73.6	62.3
*Sudden*	38.6	22.1	37.1	26.4	37.7
Attending physicians†					
*Family physician*	46.1	45.5	50.7	75.9	58.4
*Specialist*	49.6	51.9	45.1	20.0	54.3
*Other*	4.2	2.6	4.3	4.0	3.5

Percentages are weighted for representativeness.

Percentages are column percentages.

*Other illnesses within palliative subset: Causes of death from benign neoplasm, in situ neoplasms or neoplasms of uncertain or unknown behaviour (n = 50), Parkinson’s disease (n = 29), Motor neurone disease (n = 22), HIV/aids (n = 3).

†Pearson χ2 test testing for differences between the four causes of death: cancer, organ failure, dementia and other illnesses. All p-values were <.001.

### Use of palliative care services

In 29% of all deaths and 47% of all non-sudden deaths palliative care services were used (Table 2). Of those dying non-suddenly palliative care services were used in 34% of people with organ failure, 48% with dementia and 73% with cancer. Half of the patients who used palliative care were referred less than ten days before death. People with cancer were referred to any type of palliative care service earlier (median: 16 days before death) than those with organ failure (five days), dementia (eight days) or other illnesses (ten days). The differences in use of palliative care services and days between referral and death between illness groups were also significant in multivariable analyses when controlled for age, sex and place of death (not in table).

**Table 2:** Referral and time of onset of specialist palliative care services; % of all deaths (% of non-sudden deaths).

	All deaths (non-sudden deaths)	Cancer	Organ Failure	Dementia	Other illness within palliative subset	p-value
	N = 3751 (2305)	N = 919 (715)	N = 458 (288)	N = 436 (320)	N = 104 (66)	
Any type	29.1 (47.6)	56.4 (72.6)	21.1 (33.7)	35.6 (48.4)	27.6 (44.6)	<.001† (<.001†)
*Palliative care support at home*	8.9 (14.6)	25.6 (33.0)	4.2 (6.7)	4.8 (6.6)	10.5 (16.9)	<.001† (<.001†)
*Hospital-based palliative care service (excl*. *Palliative care unit)*	11.4 (18.6)	23.4 (30.1)	9.0 (14.4)	7.9 (10.7)	6.7 (10.8)	<.001† (<.001†)
*Palliative care unit*	3.8 (6.2)	11.5 (14.8)	0.7 (1.1)	0.9 (1.3)	4.8 (7.7)	<.001† (<.001†)
*Palliative care reference person in a nursing home*	8.2 (13.4)	5.0 (6.5)	8.4 (13.3)	24.5 (33.3)	9.4 (15.2)	<.001† (<.001†)
Time of onset of palliative care service*						
*Median days prior to death [p25-p75]*	10 [5–30]	16 [7–31]	5 [3–14]	8 [4–20]	10 [5–30]	<.001‡

Abbreviations: P25-75 = percentile 25 to 75.

Percentages are column percentages. Percentages may not add up to total percentage of referrals because more than one palliative care service was used in some cases.

In sudden deaths referrals to palliative care have been precluded.

Time of onset of palliative care service refers to the number of days between the first referral to any of the palliative care services and death.

*Calculations for only patients with a referral to palliative care services (only available for non-sudden deaths). Missing values for time of onset n = 139 (12.8%).

†Pearson *χ* ^2^ test testing for differences in referral between the four illness groups.

**‡**Kruskal-Wallis test testing for differences in time of onset between the four illness groups.

### Reasons for not using palliative care services

The most prevalent reason for not using palliative care according to physicians was that the patient’s palliative and supportive care needs were already being sufficiently met by existing care (56%, Table 3). Other reasons indicated by physicians included that palliative care was not or not sufficiently meaningful for the patient (26%), there was not enough time to initiate palliative care (24%), the patient did not want palliative care (6%), the family did not want palliative care (4%), palliative care was not available (1.5%) or the physician did not want to deprive the patient and/or family of hope (0.5%).

**Table 3:** Reasons given by physicians for not using palliative care services (PCS) in people with an illness indicative of palliative care need and who died non-suddenly*.

	Not using PCS (N(% within category))	Care sufficient (%)	Not meaningful(%)	Not enough time (%)	Patient did not want (%)	Family did not want (%)	Not available (%)	Not take away hope (%)
Total number (%)	N = 583 (42.0)	N = 304 (56.3)	N = 142 (26.3)	N = 127 (23.5)	N = 34 (6.3)	N = 23 (4.3)	N = 8 (1.5)	N = 3 (0.5)
Cause of death								
*Cancer*	**194 (27.4)**	**51.4**	**22.5**	**26.7**	**13.0**	**5.1**	1.1	0.6
*Organ Failure*	**189 (66.3)**	**51.4**	**24.2**	**28.1**	**4.5**	**6.2**	2.8	0.6
*Dementia*	**163 (51.4)**	**66.4**	**35.5**	**13.7**	**0.0**	**1.3**	0.7	0.0
*Other illnesses within palliative subset*	**37 (55.2)**	**62.5**	**15.6**	**28.1**	**8.6**	**2.9**	0.0	0.0
Sex								
*Male*	300 (43.0)	**50.0**	27.3	26.4	**8.3**	4.7	1.4	0.4
*Female*	282 (41.7)	**62.9**	25.3	20.5	**4.2**	3.8	1.9	0.4
Age at death, in years								
*18–64 y*	**68 (30.6)**	**31.7**	29.0	**36.5**	**12.7**	3.2	4.8	0.0
*65–84 y*	**258 (37.6)**	**55.1**	22.7	**26.3**	**8.0**	3.0	1.7	1.3
*≥ 85 y*	**256 (55.1)**	**63.5**	29.2	**17.1**	**2.9**	5.8	0.8	0.0
Place of death								
*Hospital*	**244 (41.5)**	**41.7**	25.4	**42.6**	**3.0**	**3.0**	**3.4**	0.9
*Home*	**137 (37.0)**	**64.2**	23.0	**7.3**	**20.0**	**8.9**	**0.8**	0.8
*Nursing home*	**186 (47.7)**	**68.2**	29.9	**10.3**	**1.7**	**2.3**	**0.0**	0.0
Living situation								
*Alone*	**93 (39.9)**	52.2	23.3	**29.7**	**7.8**	4.4	0.0	0.0
*In household with others*	**282 (39)**	52.4	24.3	**28.0**	**9.7**	5.8	2.4	1.2
*Institution*	**196 (49.0)**	63.0	30.4	**15.2**	**0.5**	2.2	1.1	0.0
Reporting physicians								
*Family Physician*	340 (42.8)	**67.6**	27.0	**8.7**	**8.6**	5.4	0.6	0.3
*Specialist*	238 (41.8)	**41.1**	25.8	**44.2**	**3.1**	3.1	1.8	0.9

Full response answers which physicians could indicate as a reason for not using palliative care services were respectively: the care already sufficiently addressed the patient’s palliative and supportive needs; palliative care was not meaningful or not meaningful enough; there was not enough time to initiate palliative care; patient did not want it; family did not want it; palliative care was not available; to not take away the hope of the patient and/or the family.

Abbreviations: PCS = palliative care services.

Percentages are row percentages. Percentages may not add up to 100 because more than one reasons could be indicated in some cases.

Missing values for reason not using palliative care n = 43 (7%).

*Bivariate Pearson *χ* ^2^ test testing for differences in reasons of not using palliative care services between causes of death, sex, age groups, places of death, living situations and reporting physicians. Bold denotes significant at p <.05.

### Characteristics associated with not using palliative care services and reasons for not using it

Those dying from non-cancer conditions, particularly organ failure, had higher chances of not using palliative care services than those dying from cancer (Table 4). Not using palliative care services was more likely in patients older than 85 than in those younger than 65 years. Use of palliative care services did not differ between place of death or between men and women.

**Table 4:** Reasons given by physicians for not using specialist palliative care services (PCS) controlled for cause of death, place of death, sex and age: multivariate analyses.

	Not using PCS*	Care sufficient†	Not meaningful†	Not enough time†	Patient did not want†	Family did not want†
Cause of death						
*Cancer*	Ref	Ref	Ref	Ref	Ref	Ref
*Organ failure*	**4.80 (3.42–6.74)**	0.91 (0.56–1.48)	1.12 (0.66–1.92)	0.95 (0.53–1.69)	0.51 (0.21–1.25)	1.54 (0.58–4.07)
*Dementia*	**2.68 (1.90–3.78)**	1.11 (0.66–1.88)	**1.90 (1.08–3.34)**	0.92 (0.43–1.94)	n/a	**0.18 (0.03–0.92)**
*Other illnesses within palliative subset*	**2.96 (1.76–4.98)**	1.21 (0.53–2.76)	0.73 (0.28–1.88)	1.65 (0.67–4.07)	0.64 (0.23–1.78)	**0.07 (0.09–0.62)**
Place of death						
*Hospital*	Ref	Ref	Ref	Ref	Ref	Ref
*Home*	0.97 (0.74–1.27)	**2.38 (1.49–3.80)**	0.83(0.48–1.43)	**0.10 (0.05–0.21)**	**9.74 (4.04–23.47)**	**4.43 (1.61–12.17)**
*Nursing home*	0.75 (0.53–1.05)	**2.17 (1.29–3.64)**	1.00 (0.56–1.80)	**0.17 (0.09–0.34)**	1.45 (0.35–6.02)	0.84 (0.20–3.53)
Age						
*18–64 y*	Ref	Ref	Ref	Ref	Ref	Ref
*65–84 y*	1.17 (0.83–1.64)	**2.27 (1.18–4.38)**	0.67 (0.34–1.31)	0.85 (0.42–1.72)	0.73 (0.32–1.67)	0.91 (0.14–5.77)
*≥ 85 y*	**1.88 (1.27–2.80)**	**2.50 (1.22–5.12)**	0.83 (0.40–1.71)	0.75 (0.33–1.66)	0.42 (0.16–1.12)	2.75 (0.41–18.66)
Sex						
*Male*	Ref	Ref	Ref	Ref	Ref	Ref
*Female*	0.79 (0.62–1.00)	1.43 (0.96–2.14)	0.77 (0.50–1.19)	0.92 (0.55–1.52)	0.80 (0.38–1.68)	0.75 (0.30–1.87)

Abbreviations: PCS = palliative care services; n/a = not applicable because no cases.

* Complex samples multivariate logistic regression analyses with palliative care services as dependent variable (no referral vs referral) cause of death, place of death, sex and age as independent variables.

†Complex samples multivariate logistic regression analyses with reasons for non-referral as dependent variable (indicated vs not indicated) and cause of death, place of death, sex and age as independent variables.

Bold denotes significant at p<.05.

For non-referred people who died at home, compared with those who died in hospital, physicians more often stated that the existing care was sufficient. For older patients it was more likely that their palliative care needs were considered to be sufficiently addressed than it was for younger people. Not using palliative care because it was not meaningful was more likely to be indicated in people with dementia than in those with cancer or other illnesses. The reason that there was not enough time for initiating palliative care was more likely to be mentioned for people who died in hospital than for those dying at home or in a nursing home. It was less likely for people with dementia than for those with cancer or organ failure that the family did not want palliative care. The reason that patients or family refused to initiate palliative care services was more likely to be given for people who died at home compared with those dying in hospital. There were no differences between men and women in reasons for not using palliative care.

## Discussion

Our population-based study uncovered the frequency of the reasons given for why many people with illness types indicative of palliative care needs are not using palliative care services. The most indicated reasons were that 1) care received already sufficiently addressed the patient’s palliative and supportive needs, 2) palliative care was not meaningful or not meaningful enough and 3) there was not enough time to initiate palliative care. In addition, this study revealed some striking differences between patient groups in the reasons given for not using these services: for older patients the care needs were more often said to be already sufficiently addressed than for younger people, and for patients with dementia it was more likely than for those with cancer or other illnesses that palliative care was considered as not meaningful.

### Strengths and limitations

Our study is based on a representative sample of deaths in Flanders with a high response rate (61%). The retrospective design, with death as the sampling unit, is the most suitable design to describe the circumstances shortly before death for all dying people and hence to collect population-based and generalizable information on the use and non-use of palliative care[12,22,23]. The drawback of this methodology is that memory/recall bias may be possible in some cases, because there was a one- to two-month lag between death and the sending of the questionnaire. An underestimation of the use of specific palliative care services cannot be precluded, as the attending physician will not always have knowledge about the palliative care services previously involved in another setting. For overall estimation and comparisons we believe this produces less of a problem. Our study focused only on the perceptions of physicians about the reasons for not using palliative care, excluding the equally important perspectives of patients, family and other caregivers[24].

### Comparison with other research

We found that many people who were dying from illnesses indicative of palliative care needs had not used specialist palliative care services. On the one hand this might indicate that many patients die with unmet palliative care needs while on the other it corresponds with a coordinated palliative care model in which not all patients need specialist palliative care services because their treating physicians can manage palliative care problems where they are not too complex[25]. Our study indeed found that regular or existing care already sufficiently meeting the patient’s care needs was the most frequently reported reason given for not referring to palliative care services. Remarkably, this reason was more likely to be found in older people as compared with their younger counterparts. It remains a question, of course, whether physicians may overestimate their own competence or underestimate their patients’ palliative care needs[26,27].

Another striking finding in our study was that in one third of cases palliative care was perceived as not meaningful, especially considering that we limited our study to the population dying with illnesses indicative of palliative care need, as identified in the research by Rosenwax et al[12]. We found this reason particularly in cases of people dying with dementia. Nevertheless, people with dementia have been shown to have palliative care needs similar to those of cancer patients[28–30]. It has been consistently found that symptoms such as pain or spiritual care needs are under-recognized and undertreated in dementia patients[30–32]. Our results seem to suggest the persisting need for more awareness that specialized palliative care could be of great benefit to people with dementia and their families[33].

The setting in which people are dying seems also to determine why some people are not using palliative care services. At home or in a nursing home regular or existing care was much more likely to be perceived as sufficiently addressing palliative care needs than in a hospital. This may partly reflect that those who are hospitalized are sicker and more unstable. Our study also found that it was more likely that there was not enough time to initiate palliative care in a hospital setting than elsewhere. It is known that in the last months of life a significant proportion of people are hospitalized with very acute care needs, and physicians tend to focus on these acute, potentially reversible symptoms that have prompted hospitalization[34]. In contrast with Lynn and Adamson’s model of palliative care, indicating that palliative care interventions may coexist with life-prolonging and curative treatment goals, this seems often not to be the case in practice[35]. Physicians’ perceptions of palliative care as care to be provided when no more treatment is available, concomitant with their reluctance and/or inability to predict the correct time-to-death prognosis, might be a reason why there was—in their perception—not enough time to start palliative care[36,37]. Our study, in line with previous research[6], also found that if a referral to specialist palliative care took place, this was often only shortly before death.

Although often mentioned in qualitative literature as a barrier to referral to specialist palliative care, our study shows that most physicians do not consider depriving people of hope as a reason for not referring their patients[19]. The problem of access or the availability of services, often mentioned in other studies, also did not seem to be an important reason why patients were not referred which perhaps reflects the fact that the study was performed in Belgium, where equal availability and access to palliative care services across the country is a legal requirement[38].

### Policy implications and conclusion

While the WHO and other important organizations are pleading for equal access to palliative care services for all people with chronic life-limiting illnesses, our population-based study shows that even though availability of palliative care services was not a major problem, there were still many people who did not use these services. The reasons for not using palliative care found in our study inform future health policies regarding palliative care in at least two ways. Firstly, strategies to tackle palliative care needs within the population should not only focus on specialist palliative care services but also on evaluation, stimulation and the guaranteeing of adequate palliative care skills in regular caregivers. Secondly, there is still a need to promote awareness of the benefits of early palliative care, including its potential and meaningfulness in non-cancer conditions such as dementia.
